# Insulators and imprinting from flies to mammals

**DOI:** 10.1186/1741-7007-8-104

**Published:** 2010-07-30

**Authors:** Chunhui Hou, Victor G Corces

**Affiliations:** 1 Department of Biology, Emory University, 1510 Clifton Road NE, Atlanta, GA 30322, USA

## Abstract

The nuclear factor CTCF has been shown to be necessary for the maintenance of genetic imprinting at the mammalian H19/Igf2 locus. MacDonald and colleagues now report in *BMC Biology *that the mechanisms responsible for maintaining the imprinted state in *Drosophila *may be evolutionarily conserved and that CTCF may also play a critical role in this process.

See research article http://www.biomedcentral.com/1741-7007/8/105

## 

Genetic imprinting is an epigenetic phenomenon that results in the expression of certain genes in a parent-of-origin chromosome-specific manner. Although imprinting was originally discovered in insects [1] and has also been described in plants [2] and zebrafish, the phenomenon has been most widely studied in mammals [3,4]. Imprinting, resulting in the functional non-equivalence of the maternal and paternal genomes, affects the expression of developmentally important genes in mice and humans, and alterations of the process result in cancer and various genetic diseases. This is not the case in insects; gynogenic and androgenic flies (containing only maternal or paternal genomes, respectively) are perfectly viable. The molecular mechanisms controlling the establishment of imprinting are not fully understood, but those involved in the maintenance of the imprinted state have been analyzed in detail for some mammalian genes, such as the mouse locus that includes the insulin-like growth factor gene (*Igf2*) and the RNA gene *H19*. However, understanding of the mechanisms controlling imprinting in non-mammalian species has lagged behind and it is unclear whether imprinting in insects and mammals is a conserved biological process with the same underlying molecular mechanisms. In this issue of *BMC Biology*, Lloyd, Meller and colleagues [5] examine the potential role of the *Drosophila *CCCTC binding factor (dCTCF) protein in the maintenance of maternal imprinting and propose that dCTCF has an evolutionarily conserved role in the maintenance of the imprinted state.

All but one case of imprinting described in *Drosophila *is associated with position-effect variegation, in which chromosomal rearrangements place genes with visible phenotypes close to heterochromatin. MacDonald *et al. *[5] use a *Drosophila *mini-chromosome in which most of the X chromosome is deleted and the *garnet *gene is placed next to the centromeric heterochromatin; the garnet protein resembles clathrin and nonclathrin adaptin proteins and is similar to the delta subunit of the mammalian AP-3 adaptin complex. The rearrangement causes a variegated expression of *garnet*, such that the eyes of the adult flies show sectors of expressing and non-expressing cells characteristic of heterochromatin-induced silencing. Interestingly, this variegated expression is imprinted and it is observed in individuals carrying the paternal mini-X-chromosome, whereas the maternally transmitted copy shows normal expression of the *garnet *gene [6]. This observation suggests that the maternally inherited mini-X-chromosome carries an imprint established in the germline that interferes with the somatic spreading of heterochromatin silencing in the next generation.

To study whether dCTCF is involved in the differential regulation of *garnet *gene expression in the maternal versus paternal chromosomes, the authors [5] examined the effect of mutations in the dCTCF gene. Flies carrying one mutant copy of dCTCF showed reduced RNA levels (30 to 40% of that found in wild type). However, this mild reduction is sufficient to significantly compromise expression of the *garnet *gene from the maternal mini-X-chromosome, leading to a variegated eye color similar to that seen from the paternally inherited chromosome. This observation suggests that dCTCF is required for the non-variegated expression of the *garnet *gene when it is maternally inherited.

Two different processes affect the visually observed *garnet *phenotype: the transcription of the gene under the control of regulatory sequences and the spreading of heterochromatin silencing - these two components may not be easy to separate mechanistically. In the mammalian *H19*/*Igf2 *locus, direct transcriptional activation can be distinguished from indirect effects from the surrounding chromatin thanks to information obtained from the analysis of the function of CTCF in the spatial organization of the maternal locus. This organization functions to maintain the imprinted expression of *H19 *and the silencing of *Igf2 *on the maternal chromosome. If CTCF binding in the imprinting control region (ICR) is abolished, both *Igf2 *and *H19 *can be transcribed from the maternal allele because of the disruption in the CTCF-mediated maternal allele chromatin organization; this organization prevents the interaction between enhancers and the promoter of *Igf2. *These observations suggest that the role of CTCF in the maintenance of imprinting involves its ability to mediate interactions that result in a specific three-dimensional architecture of the locus. In fact, CTCF is not directly involved in the transcription activation of the mouse *H19 *and *Igf2 *genes [7-10].

In *Drosophila*, the primary effect of the maternal imprint is to inhibit the spread of heterochromatin-induced silencing (that is, silencing that turns euchromatin into a more compact state that limits the access of transcription factors to the genes). The finding that the *garnet *gene is poorly expressed from the paternally transmitted mini-X-chromosome and is not affected by reduced dCTCF expression [5] suggests that heterochromatinization is an effective gene silencing mechanism. Expression of the *garnet *gene in the maternally derived mini-X-chromosome could then be accomplished by the establishment of a distinct barrier to the spread of heterochromatin (Figure 1a) or by a direct effect of dCTCF on *garnet *transcription that indirectly antagonizes heterochromatin spreading (Figure 1b). In the latter case, an epigenetically transmitted increased expression of *garnet *in the maternal germline would be the imprint that inhibits heterochromatin spreading in the somatic cells of the progeny.

**Figure 1:**
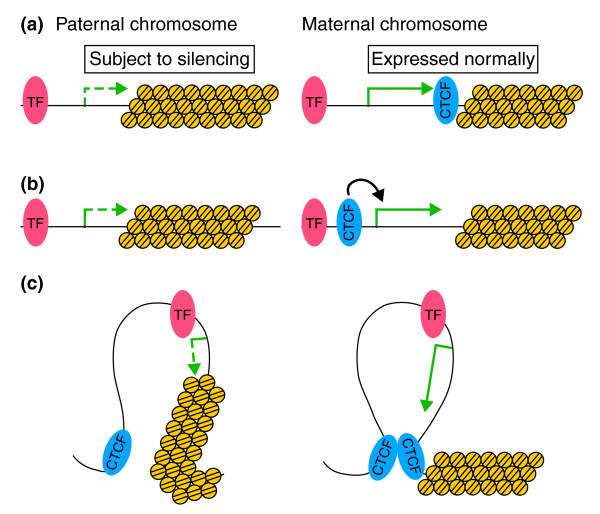
**Possible models to explain the role of dCTCF in the maintenance of imprinting in *Drosophila***. In all panels, a hypothetical transcription factor (TF) controlling the expression of the *garnet *gene is shown as a red oval; the *garnet *gene is represented as a green arrow, which is dashed when the gene is subject to silencing by heterochromatin and solid when it is expressed normally; dCTCF is represented by a blue oval; nucleosomes are shown as yellow circles; and DNA is in black. **(a) **dCTCF in the maternal chromosome forms a barrier against the spreading of heterochromatin, leading to normal expression of the adjacent gene; presumably, CTCF is not present in the paternal chromosome and heterochromatin spreads into the gene. **(b) **dCTCF, either directly or in combination with other factors, affects the transcription of the *garnet *gene, antagonizing the spreading of heterochromatin and overcoming its silencing effect. **(c) **An alternative explanation that involves the formation of a loop between a dCTCF site adjacent to the heterochromatin and a second site somewhere else in the genome. The *garnet *gene and its regulatory sequences are located inside of the loop, which protects the gene against heterochromatin silencing. The models in (a,c) are conceptually similar but mechanistically different and the latter is more in line with observations in mammals.

After showing a role for dCTCF in the maintenance of the maternal imprint, MacDonald *et al. *[5] explored the possibility of a similar function for this protein in the establishment of the imprinted state in the maternal germline. This process is poorly understood, not only in insects but also in mammals. Although the exact nature of the proteins involved and how they function in the establishment of genetic imprinting during gametogenisis are unknown, some candidate proteins have been excluded from a direct role in this process. For example, it is now clear that CTCF is not necessary for the establishment of imprinting in the mouse *H19*/*Igf2 *locus. Given the functional conservation of CTCF as an insulator protein between flies and mammals, it is interesting to ask whether this is also the case in *Drosophila*. MacDonald *et al. *[5] found that expression of the *garnet *gene is not subject to heterochromatin-induced silencing in a mini-X-chromosome inherited from females heterozygous for mutations in the dCTCF gene. This observation suggests that dCTCF is not required for the establishment of the maternal imprint. However, the question remains as to whether further reduction in the levels of dCTCF in the maternal germline may actually show an effect on this process. For example, it is possible that dCTCF expression during oogenesis is much higher than in somatic cells, and that the small reduction in dCTCF levels in heterozygous mutant females is not sufficient to affect the establishment of the imprint. Alternatively, there may be other mechanisms that can prevent the spreading of heterochromatin during gametogenesis independent of dCTCF. Such a mechanism could use other insulator proteins or alternative processes to prevent heterochromatin spreading.

CTCF has multiple roles in chromatin organization and gene regulation that derive from its ability to mediate intra- and inter-chromosome interactions [11]. Given that the ability of CTCF to organize chromatin resulting from its insulator function is the basis for its role in transcriptional regulation and genetic imprinting, many of these functions are probably evolutionarily conserved from flies to humans. In vertebrates, CTCF has been shown to act as an enhancer-blocking insulator and to function by creating intra- and inter-chromosomal loops, but CTCF does not seem to form barrier insulators, which seem to function by recruiting chromatin remodeling proteins that act by covalently modifying histones. Although such a distinction has not yet been made in *Drosophila*, it is possible that the role of dCTCF in maintaining the imprinted state is related to the ability of this protein to mediate interactions that create a chromatin domain insulated from heterochromatin silencing (Figure 1c). The findings of MacDonald *et al. *[5] agree with this idea. Additional analysis of the molecular mechanism of imprinting in *Drosophila *will shed new light not only on the understanding of the mechanisms controlling this process, but also on the understanding of the evolutionary conservation of CTCF function.
